# Supplementary material for: From resilience to satisfaction: Defining supply chain solutions for agri-food SMEs through quality approach
Source: PLoS One. 2022 Feb 2;17(2):e0263393. doi: 10.1371/journal.pone.0263393 (PMC8809543; doi:10.1371/journal.pone.0263393)
Supplement: S2 File — (DOCX) [file pone.0263393.s002.docx]

**IN-DEPTH INTERVIEW SUMMARY RESULT

Note: All raw data is obtained in Indonesian, as the 1st author is a native Indonesian, which is then translated into English.**

**Translation customer opinion summary into attributes of customer needs.**

| the texture is not mushy... the color is not pale... the smell is fresh... it is not putrid... the condition of the product is clean... a wide variety of product... the stock is always available... it tastes delicious... the price can be stable even in a pandemic... the display between products is not mixed, so there is no contamination... the shape is not deformed... the color image is pleasing to the eye... the texture is firm and not rotten... no foul smell... the price does not go up... the taste is good and fresh... not mushy... always available when needed. there is a wide variety of agricultural products so it does not get boring... the price remains affordable... the quality of the product is of attractive colors and textures that are not rotten. |
| --- |

Cleanliness

Stock availability

Fresh smell

Firm texture

F

Contamination-free

Tastiness

Proper shape

Product variation

Attractive bright colour

Price stability

**Translation industry actors’ opinion summary related to agri-food risk into supply chain risks attributes.**

| Failure to harvest... Damage to equipment due to age of equipment... A great flood that damaged the crops... trapped... Not sure how to deal with a major flood...risk of being attacked by pests... livestock diseases... poor storage conditions. Not knowing if the packaging is in poor condition... Transportation and production facilities that do not work... Demanding information from retailers that does not match sales facts... Many workers here do not have higher education so they do not really understand management... Shipping accidents... Robbed on the road... Traffic jams especially in big cities... Inadequate warehouse space... Theft or robbery of logistics vehicles... Vehicles that are old and do not work properly... Some workers do not know how to use tools properly and correctly... Death of livestock on a large scale... Delivery that likes to be late due to traffic jams... crops are not suitable... Lack of coordination, especially with farmers and small intermediaries. |
| --- |

| Criminal activities  Bullwhip effect  Human resource risk  Traffic congestion  Equipment failure    Transportation accident  Harvest failure  F  Improper storage |
| --- |

**Translation industry actors’ opinion summary related to supply chain resilience into resilience action attributes.**

There must be a routine maintenance plan... the use of IT technology, especially for information coordination... Increasing understanding in terms of quality standards... Having an accurate plan for forecasting supply... Planning to anticipate natural disasters that may disrupt crops... Maintain cleanliness and health of livestock on a regular basis... Collaboration between organizations to improve quality... Training workers on the importance of maintaining quality and how to maintain it... Training industry players on how to accurately forecast demand and supply... Making plans in terms of product varieties and planting years... Establish coordination and transparency between organizational levels in the supply chain... Strive for the best quality across all organizations... Coordinate with each other to provide relevant information that can make the shared supply chain vision a success.

Supply chain coordination

Continuous Training

Preventive Maintenance

Disaster Prevention Plan

F

Forecasting supply chain

IT Utilization

Continuous Training

Continuous Training
