# Supplementary material for: From resilience to satisfaction: Defining supply chain solutions for agri-food SMEs through quality approach
Source: PLoS One. 2022 Feb 2;17(2):e0263393. doi: 10.1371/journal.pone.0263393 (PMC8809543; doi:10.1371/journal.pone.0263393)
Supplement: S3 File — (DOCX) [file pone.0263393.s003.docx]

**Questionnaire of customer needs in agri-food SMEs industry**

Dear customers:

This questionnaire is designed to identify customer needs related to the agri-food industry. We are asking you to complete or respond to this questionnaire in order to understand customer needs and, based on this, identify the risks that will impact the industry and determine actions that industry players can take to mitigate the risks and meet the needs of these customers. This survey will be used for academic purposes only and is anonymous. We greatly appreciate your participation in this survey and thank you.

Ph.D. program of Economics,

Hungarian University of Agriculture and Life Sciences

Tutur Wicaksono

Csaba Balint Illes

**DEMOGRAPHIC INFORMATION**

1. Name (optional):

2. Gender:

3. Age:

4. Education:
5. Purchasing Experience:

6. Purchasing Frequency:

**IMPORTANCE LEVEL OF CUSTOMER NEEDS ATTRIBUTES**

| **Scale** | **Value** |
| --- | --- |
| Very Important | 5 |
| Important | 4 |
| Moderately Important | 3 |
| Unimportant | 2 |
| Least Important | 1 |

| Attributes list | Importance Value | | | | |
| --- | --- | --- | --- | --- | --- |
|  | 1 | 2 | 3 | 4 | 5 |
| Firm texture |  |  |  |  |  |
| Fresh smell |  |  |  |  |  |
| Attractive bright colour |  |  |  |  |  |
| Stock availability |  |  |  |  |  |
| Cleanliness |  |  |  |  |  |
| Proper shape |  |  |  |  |  |
| Tastiness |  |  |  |  |  |
| Contamination-free |  |  |  |  |  |
| Price stability |  |  |  |  |  |
| Product variation |  |  |  |  |  |
